# Where Are We With Human Lice? A Review of the Current State of Knowledge

**DOI:** 10.3389/fcimb.2019.00474

**Published:** 2020-01-21

**Authors:** Nadia Amanzougaghene, Florence Fenollar, Didier Raoult, Oleg Mediannikov

**Affiliations:** ^1^Aix Marseille Univ, IRD, AP-HM, SSA, VITROME, Marseille, France; ^2^IHU-Méditerranée Infection, Marseille, France; ^3^Aix Marseille Univ, IRD, AP-HM, MEPHI, Marseille, France

**Keywords:** *Pediculus humanus*, biology, epidemiology, phylogeny, disease-vector, control

## Abstract

*Pediculus humanus* is an obligate bloodsucking ectoparasite of human that includes two ecotypes, head louse and body louse, which differ slightly in morphology and biology, but have distinct ecologies. Phylogenetically, they are classified on six mitochondrial clades (A, B, C, D, E, and F), head louse encompasses the full genetic diversity of clades, while body louse belongs to clades A and D. Recent studies suggested that not only body louse, but also head louse can transmit disease, which warrants greater attention as a serious public health problem. The recent sequencing of body louse genome confirmed that *P. humanus* has the smallest genome of any hemimetabolous insect reported to date, and also revealed numerous interesting characteristics in the nuclear and mitochondrial genomes. The transcriptome analyses showed that body and head lice were almost genetically identical. Indeed, the phenotypic flexibility associated with the emergence of body lice, is probably a result of regulatory changes, perhaps epigenetic in origin, triggered by environmental signals. Current lice control strategies have proven unsuccessful. For instance, ivermectin represents a relatively new and very promising pediculicide. However, ivermectin resistance in the field has begun to be reported. Therefore, novel opportunities for pest control strategies are needed. Our objective here is to review the current state of knowledge on the biology, epidemiology, phylogeny, disease-vector and control of this fascinating and very intimate human parasite.

## Biology, Epidemiology and Genomic of Lice

Human lice, commonly known as sucking lice, are hemimetabolous insects that belong to the suborder *Anoplura* (order: *Phthiraptera*). They pierce the skin of their human hosts and feed on their blood, which is their only diet (Durden and Musser, 1994). More than 530 species have been described and each species parasitizes one or more closely related placental mammalian host species (Durden and Musser, 1994). Two louse species are known to parasite humans, *Pediculus humanus* and *Pthirus pubis* (Reed et al., 2007). *Pthirus pubis*, found in the pubic area and known as the pubic or crab louse (not considered further in this review) belongs to the genus *Pthirus*, which is shared with gorillas (*Pt. gorilla*) (Durden and Musser, 1994; Reed et al., 2007). *Pediculus humanus* belongs to the genus of *Pediculus*, which is shared with chimpanzees (*P. schaeffi*) and New World monkey (*P. mjobergi*) (Durden and Musser, 1994; Reed et al., 2007; Drali et al., 2016a).

*Pediculus humanus* comprises two ecotypes, which are body lice (*P. h. humanus*) and head lice (*P. h. capitis*). These two ecotypes have almost the same morphology but differ in their ecology and have distinct nutritional patterns (Veracx and Raoult, 2012). The head louse lives, breeds, and lays its eggs (nits) at the base of hair shafts and frequently feed on human blood every 4–6 h (Light et al., 2008b; Veracx and Raoult, 2012). Body louse lives and lays eggs in clothing, feeds less frequently and ingests greater quantities compared with head louse (Light et al., 2008b; Li et al., 2010; Brouqui, 2011). In addition, it lays more eggs and grows faster than head louse (Light et al., 2008b; Li et al., 2010). Furthermore, body louse is more resistant to environmental conditions, can withstand lower humidity and survives longer outside the host (more than 72 h for the off-host survival) (Veracx and Raoult, 2012).

Head louse infestation is very common worldwide, especially among schoolchildren, whatever their hygiene status, and the transmission occurs mainly by head-to-head contact (Chosidow, 2000). Adults with poor personal hygiene are also commonly affected (Chosidow, 2000). Body louse infestation is less prevalent and is related with poor hygiene and a lack of sanitation, overcrowding, damp, and cold weather conditions (Raoult and Roux, 1999). For that reason, homeless, jail, and refugee populations are predominantly affected (Raoult and Roux, 1999; Brouqui, 2011). In addition to their role as a dangerous disease vector (we will discuss this point in greater detail later in the review), louse infestations cause itching that may lead to intense irritation (Chosidow, 2000; Veracx and Raoult, 2012). Severe itching can lead to excoriations in which a secondary bacterial infection is likely to occur (Chosidow, 2000). Post inflammatory pigmentation is also common in chronically infested persons (Chosidow, 2000).

The recent sequencing and annotation of body louse genome confirmed that *P. humanus* harbor the smallest known holometabolic insect genome sequenced to date, and revealed interesting information and characteristics on nuclear and mitochondrial genomes. Thus, body louse genome holds great potential for the understanding of the coevolution among lice population, symbionts and pathogens (Kirkness et al., 2010). Its genome about 108 Mb contains 10,773 predicted protein-coding genes and 57 microRNAs (Kirkness et al., 2010). Interestingly, the number of genes related to sensing and responding to the environmental factors were significantly reduced in body louse genome compared to other insect genomes (Kirkness et al., 2010; Pittendrigh et al., 2013). Thus, reflecting its simple life cycle, whereby humans serve as the sole host and human blood as the only diet (Kirkness et al., 2010; Pittendrigh et al., 2013).

Unlike bilateral animals from which the 37 mitochondrial genes are typically arranged on a single circular chromosome, the body and head lice have their genes organized in an unusual architecture of 20 minichromosomes, each minichromosome has a size of 3–4 kb and has 1–3 genes and a control region (Shao et al., 2012). Notably, this extensively fragmented mitochondrial genome of *P. humanus* is a fascinating phenomenon and represents the most radical departure to date in bilateral animals, and may be associated with the loss of the gene involved in mitochondrial genome replication which encodes the mitochondrial single-stranded DNA binding protein (Kirkness et al., 2010; Shao et al., 2012). However, why and how exactly mitochondrial genome became fragmented, are still poorly understood.

Body and head lice host the same primary endosymbiotic bacteria (*Candidatus* Riesia pediculicola) that supply the lice with B-vitamins, absent in the human blood (Kirkness et al., 2010; Boyd and Reed, 2012). *Candidatus* Riesia pediculicola has a small genome that encodes <600 genes distributed between a short linear chromosome (~0.57 Mb) and a circular plasmid (~8 kb) including genes required for the synthesis of essential B vitamins (Kirkness et al., 2010; Boyd et al., 2017). The bacterium is housed in specialized structures known as mycetomes, localized on the ventral side of the midgut, and is vertically transmitted from the female louse to its progeny (Perotti et al., 2007). It belongs to the family of *Enterobacteriaceae* within the *Candidatus* Riesia genus, which is shared with lice that parasitize chimpanzees and gorillas (Perotti et al., 2007; Boyd et al., 2017). Phylogenetic studies have shown that the symbiont co-evolved with head and body lice and shared a common ancestor with *P. schaeffi* endosymbiont (*Candidatus* Riesa pediculischaeffi) roughly 5.4 Mya ago (Boyd et al., 2017). Lice endosymbionts might also support investigations on human evolution (Boyd et al., 2017).

Moreover, in addition to being fundamental to lice development and survival, which makes it an interesting target for the development of an alternative lice control strategy (we will discuss this point in detail later in the review), the question of whether this symbiont has an influence on lice behavior or competence as a disease vector merits further study.

## Phylogeny and Phylogeography of Lice

### Phylogeny Relationships Between Head and Body Lice

The body and head lice have a morphology and biological characteristics almost similar, but differ in their ecological niches (Veracx and Raoult, 2012). Although the taxonomic status of these two types of lice has been debated for more than a century, they are now considered as ecotypes of a single species as opposed to distinct species (Light et al., 2008b; Veracx and Raoult, 2012; Tovar-Corona et al., 2015). Despite numerous studies, the genetic basis and evolutionary relationships among body and head lice remain obscure.

Until recently, the most predominant opinion was that body louse descended from head louse in nature (Veracx and Raoult, 2012). Indeed, as the body louse lives and lays eggs on the clothing of the host (Raoult and Roux, 1999; Light et al., 2008b; Veracx and Raoult, 2012), it was thought that body lice had only recently appeared when modern humans started wearing clothes (Kittler et al., 2003; Light et al., 2008b). However, the most recent data available do not share the same view. Indeed, a novel theory for the emergence of body lice has been reported recently, suggesting that under certain conditions of poor hygiene, an infestation of head lice can turn into a massive infestation, which has allowed variants (genetic or phenotypic) of head lice able of ingesting a large amount of blood, a typical characteristic of body lice, to colonize clothing (Li et al., 2010; Brouqui, 2011; Veracx and Raoult, 2012). This assumption was based on the genotypic and phylogenetic analyses using highly variable intergenic spacers showing that head and body lice are not indistinguishable (Li et al., 2010). In addition, several researchers pointed that when head lice raised under appropriate conditions, they could develop into body louse ecotypes (Keilin and Nuttall, 1919; Alpatov and Nastukova, 1955). Thus, the divergence of head and body lice is obviously not the result of a single event, but probably takes place constantly among the two shared louse clades A and D (see below for louse mitochondrial clades), this transformation being facilitated by mass infestations (Li et al., 2010; Veracx and Raoult, 2012).

Moreover, the comparison of head and body louse transcriptomes has revealed that among the 14 genes with differential expression, only one gene was thought to be missing in head louse, which is the PHUM540560 gene that encodes a hypothetical 69-amino acids protein of unknown function (Olds et al., 2012). A thorough analysis conducted by Drali et al. (2013) revealed that this gene was also present in the head louse but with a rearranged sequence compared to body louse. Notably, based on the variation of this gene, a molecular tool based on real-time PCR has been developed and allows for the first time to differentiate between the two ecotypes (Drali et al., 2013). These findings indicate that head and body lice have almost the same genomic content; therefore, their phenotypic difference is likely to be associated with a variation in gene expression (Olds et al., 2012; Veracx and Raoult, 2012).

Indeed, a recent study analyzed alternative splicing using previously reported transcriptome data for both head and body lice, and reported evidence of differences between transcription pools (Tovar-Corona et al., 2015). Interestingly, while no genes functional categories associated with louse-specific alternative splicing events were found to be overexpressed, genes containing body louse-specific alternative splicing events were found to be significantly enriched for functional categories associated with development of the nervous system and feeding, and ovarian follicle cells, as well as regulation of transcription (Tovar-Corona et al., 2015). For example, the observed enrichment of functional categories related to feeding, in particular the development of salivary gland and open tracheal system, may reflect the adaptation of body lice to more sporadic dietary habits in the clothing environment compared with the more continuous feeding of head lice (Tovar-Corona et al., 2015). Alternative splicing is through to be a major contributor to proteome diversification (Nakka et al., 2018). Indeed, several studies have strongly associated alternative splicing with phenotypic plasticity in eusocial insects, where distinct regulatory programs and phenotypes derive from a single genome (Lyko et al., 2010; Terrapon et al., 2014). Thus, the changes in the development program observed in body lice compared to head lice, may underpin some of the phenotypic flexibility observed in body lice allowing them to colonize clothing (Tovar-Corona et al., 2015).

Taken together, these data evidence that the phenotypic shifts associated with the emergence of body lice are likely to be a consequence of regulatory changes, possibly epigenetic in origin, triggered by environmental cues. Such phenotypic modification has been reported to occur in other insects, such as in honey bees, termites, and migratory locusts (Simpson et al., 2001; Hayashi et al., 2007; Lyko et al., 2010). For example, in honey bees, the development of queens and workers is strictly controlled by the differential feeding of royal jelly and their adult behaviors are accompanied by epigenomic changes (Lyko et al., 2010). Certainly, further efforts on the genetic studies of head and body lice are needed to link their genetic difference with phenotypic differences. Whole genome sequencing of head lice and comparative genomics combined with transcriptomic, proteomic, and epigenomic studies between head and body lice would be useful to better understand lice and address these questions.

### Louse Mitochondrial Clades

**Figure 1:**
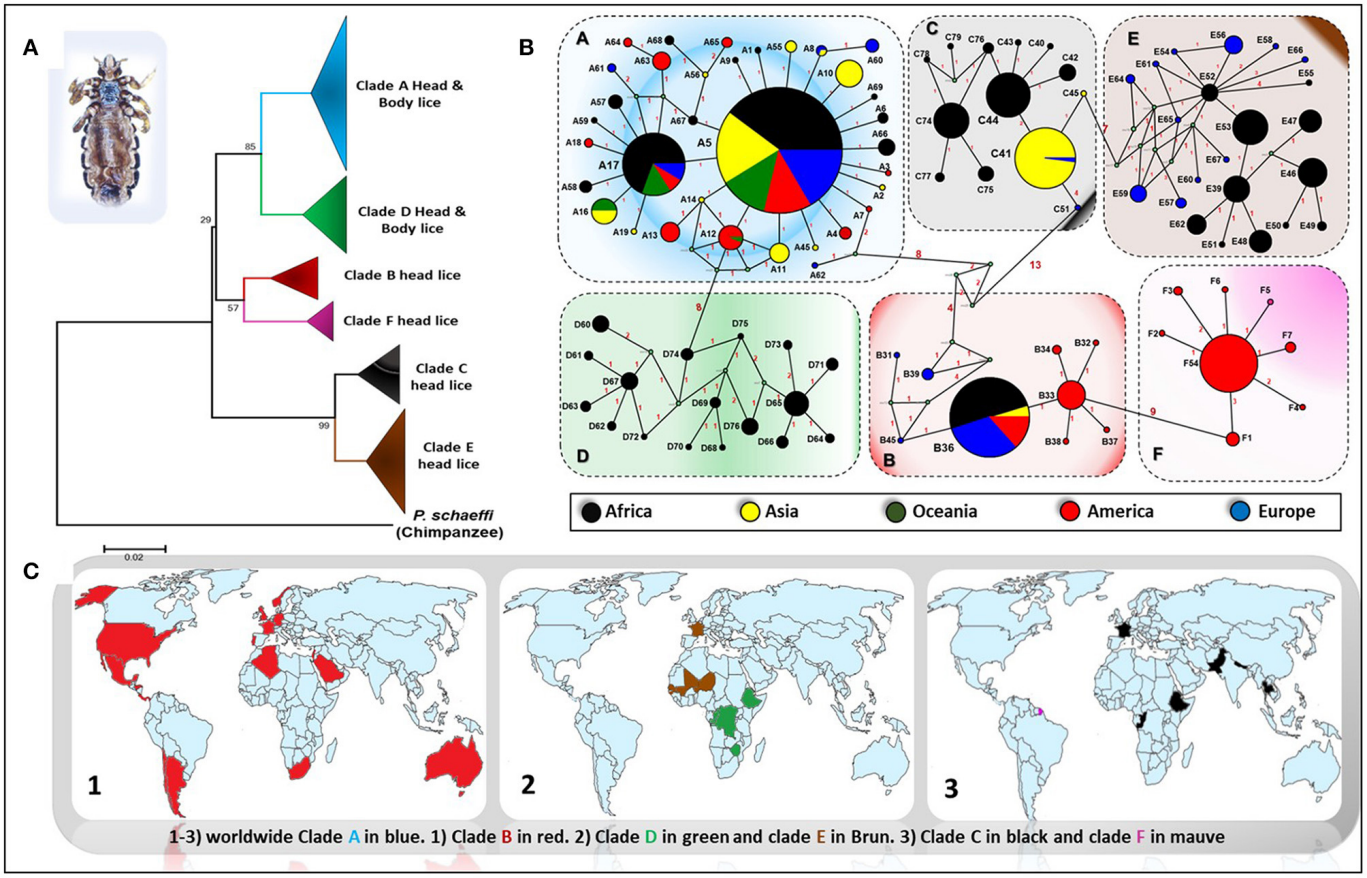
Phylogeography of body and head lice haplogroups. **(A)** Neighbor-joining tree based on Cytb haplotypes. **(B)** Median Joining Network representing the existing relationships between different haplotypes. Pie colors and sizes in circles represent the continents and the number of their sequence for a haplotype. **(C)** Maps of the world showing the distribution of louse clades.

Genetic studies based on mitochondrial genes have inferred to *Pediculus* lice a robust phylogenetic classification into several divergent clades or haplogroups, exhibiting some geographic differences (Figure 1) (Light et al., 2008b; Ashfaq et al., 2015; Drali et al., 2015b; Amanzougaghene et al., 2017). Six mitochondrial clades were described (A, D, B, F, C, and E) (Ashfaq et al., 2015; Drali et al., 2015b; Amanzougaghene et al., 2016b, 2017, 2019). In addition to the inter clade diversity, human lice also display intra clade diversity, as illustrated by the multiple distinct haplotypes for each clade (Figure 1B) (Ascunce et al., 2013; Ashfaq et al., 2015; Amanzougaghene et al., 2016a; Drali et al., 2016a). Head lice encompass all clades diversity, while body lice belong to clades A and D only (Amanzougaghene et al., 2017).

Clade A is widely and globally distributed, while clade D has so far only been reported in sub-Saharan African countries, including the Democratic Republic of Congo (DRC) and the Republic of Congo (Congo-Brazzaville), where it was characterized for the first time among the Pygmy populations (Ascunce et al., 2013; Ashfaq et al., 2015; Drali et al., 2015b; Amanzougaghene et al., 2016a). The clade D was also found in Zimbabwe and Ethiopia (Amanzougaghene et al., 2016a, 2019). Clade B is mainly found in America, where it exhibits a high diversification (Reed et al., 2004; Light et al., 2008a; Ascunce et al., 2013). This clade is also found in Western Europe, Australia, North Algeria, South Africa, Saudi Arabia, and in Israel among head lice remains dating back to the Roman period (Raoult et al., 2008; Ascunce et al., 2013; Ashfaq et al., 2015; Boutellis et al., 2015; Al-Shahrani et al., 2017). Clade F is the sister group of clade B. This clade was first described in head lice recovered from members of the Wayampi community living in a remote Trois-Sauts village, located in French Guiana. This clade was also found in Argentina and Mexico (Amanzougaghene et al., 2019). *Pediculus mjobergi*, the lice of South American monkeys of *Cebidae* family, also belong to this clade. These lice are supposed to be acquired by their monkey's hosts from the first peoples who penetrated the New World across the Bering straits thousands of years ago (Drali et al., 2016a; Amanzougaghene et al., 2019).

Clade C has been reported mainly from two continents, Africa (from Ethiopia and the Republic of Congo) and Asia (Nepal, Pakistan, and Thailand) (Sunantaraporn et al., 2015; Amanzougaghene et al., 2016a, 2017). Lastly, clade E was chiefly found in head lice from West African countries (in Senegal and Mali) (Amanzougaghene et al., 2017, 2019). This clade was also frequently recovered from migrants communities coming mainly from West African countries, as is the case of its detection among Nigerian refugees in Algeria and from migrant communities living in Bobigny, France (Candy et al., 2018a; Louni et al., 2018). Taken together, these data support the idea that all these louse clades accompanied our hominid ancestors since their migrations out of Africa (Li et al., 2010; Amanzougaghene et al., 2017).

### Lice as Marker of Human Evolution

Because human lice are highly host specific and have been evolving in tandem with their primate hosts for a thousand of years, they offer a unique feature to reconstruct human migration and human evolutionary history, thereby complementing the hominin fossil records (Reed et al., 2004; Boutellis et al., 2014; Perry, 2014). Studies to date have only started to exploit this invaluable source of precious information (Perry, 2014). Indeed, phylogenetic analyses of *Pediculus* lice have confirmed some events in the human evolution. For instance, lice of human (*P. humanus*) and those of chimpanzee (*P. schaeffi*) have shared a common ancestor about 6 million years ago, which appears to be meeting to the estimated date for the divergence of their respective hosts (Reed et al., 2004, 2007). The lice population presents the genetic signature of a recent demographic expansion that occurred roughly 100,000 years ago, coinciding with the out-of-Africa expansion of modern human hosts, allowing us to use lice to learn about human migration, such as the travel itinerary and the timing of peopling the New Word (Reed et al., 2004; Ascunce et al., 2013). Reed et al. (2004) suggested that two ancient head louse clades (B and C), which originated before modern *Homo sapiens*, were from archaic hominins (Reed et al., 2004). They found that clade B head lice diverged from clade A between 0.7 and 1.2 Mya, and may have evolved on Neanderthals populated the Eurasian continent, whereas clade C is even more ancient (ca. 2 Mya) and may have evolved on *H. erectus* (Reed et al., 2004). Because head lice are primarily transmitted horizontally through a physical contact host-to-host, these data support the view of the existence of a direct contact of modern humans with archaic hominin species, and from which they have acquired a distinct lineages of head lice (Reed et al., 2004).

Lice have also helped to elucidating events in the history of human evolution that are absent or uncertain from host fossil or host DNA (Raoult et al., 2008), such as the date on which *H. sapiens* started wearing clothing by estimating the age of onset of the body louse, which would have evolved only after humans had worn these clothes since this louse lives exclusively in clothing (Kittler et al., 2003). As recently suggested by Toups et al. (2011), based on the molecular dating of body louse, the use of clothing would have started between 83,000 and 170,000 years previous to what was expected (anywhere from 40 Ka to 3 Ma based on the emergence of eyed needles and the loss of body hair), and the use of clothing by *H. sapiens* probably originated in Africa and may have made it easier for them to move during frosty weathers as they traveled outside Africa and possibly around the planet earth (Toups et al., 2011).

All these studies demonstrate how lice can provide us with information about the mysteries of our evolution. Further studies on lice and other specific human host parasites, which carry a written record of our past in their DNA, will clarify other events of the human history, while extinct hominid species no longer exist to give us clues about our origins.

### Paleoentomology

Paleoentomological investigations can provide relevant information on the antiquity of lice and their relationship with humans, ancient human migrations across the world and their sanitary conditions (Amanzougaghene et al., 2016b; Drali et al., 2016b). Human lice are most likely among the oldest permanent ectoparasites of humans (Mumcuoglu, 2008; Boutellis et al., 2014). In the Bible, louse infestations are described as the third plague (Mumcuoglu, 2008). Louse infestations of the ancient inhabitants of the Middle East have also been evidenced from the Sumerian, Akkadian, and Egyptian sources (Mumcuoglu, 2008). Moreover, the fossil record of lice and nits from different archaeological sites around the world has expanded greatly over the last 20 years (Drali et al., 2016b).

The oldest head louse nits were found on hair sample collected from an archaic human skeleton found in an archeological site in northeastern Brazil (Araújo et al., 2000). These samples were dated approximately to more than 10,000 years old, demonstrating that the lice were introduced into the New World through the earliest peoples (Araújo et al., 2000). In the Old World, the oldest head louse remains were recovered from an individual who lived in the Nahal Hemar cave near the Dead Sea in Israel during the Neolithic era, 9,000 years ago (Mumcuoglu, 2008). Head lice and their eggs have been recovered on mummified remains in Egypt, China, the Aleutian Islands, Greenland and South America (Drali et al., 2016b). Head lice combs from about 6,500 years ago, very similar to modern lice combs, were already used for delousing in the Pharaonic era in Egypt (Mumcuoglu, 2008). Head lice and their nits have been recovered in combs from different archaeological excavations sites in Egypt and Israel (Mumcuoglu, 2008; Drali et al., 2015a, 2016b; Amanzougaghene et al., 2016b).

The DNA analysis conducted on ancient louse samples provided valuable information to record past genetic structure and diversity of *Pediculus* lice that can be exploited to reconstruct ancient human evolutionary events (Amanzougaghene et al., 2016b; Drali et al., 2016b). However, so far, only a few genetic studies have focused on the remains of ancient lice. Thus, phylogenetic analysis of the remains of head lice eggs dating from the Chalcolithic and early Islamic periods found in Israel has shown that these remains can be associated with people from West Africa because they belonged to the mitochondrial subclade C (now known as clade E) specific to this region (Drali et al., 2015a). The analysis of pre-Columbian mummies' lice revealed that head lice belonged to clade A, had a pre-Columbian presence in the America, and would therefore be linked to the Old World (Raoult et al., 2008). Subsequent study conducted by Boutellis et al. (2013a) endorsed this finding and further reported that the presence of clade B head lice on the American continent dates back more than 4,000 years, before the arrival of European settlers, which support an American origin for this clade (Boutellis et al., 2013a). However, this hypothesis has been challenged by its recent discovery among the remains of head lice from 2,000 years ago, found in Israel, supporting a Middle Eastern origin for this clade, followed by its introduction to the American continent with the first people who set up there (Amanzougaghene et al., 2016b).

## Lice-Borne Bacterial Disease

### Body Louse Associated Pathogens

**Figure 2:**
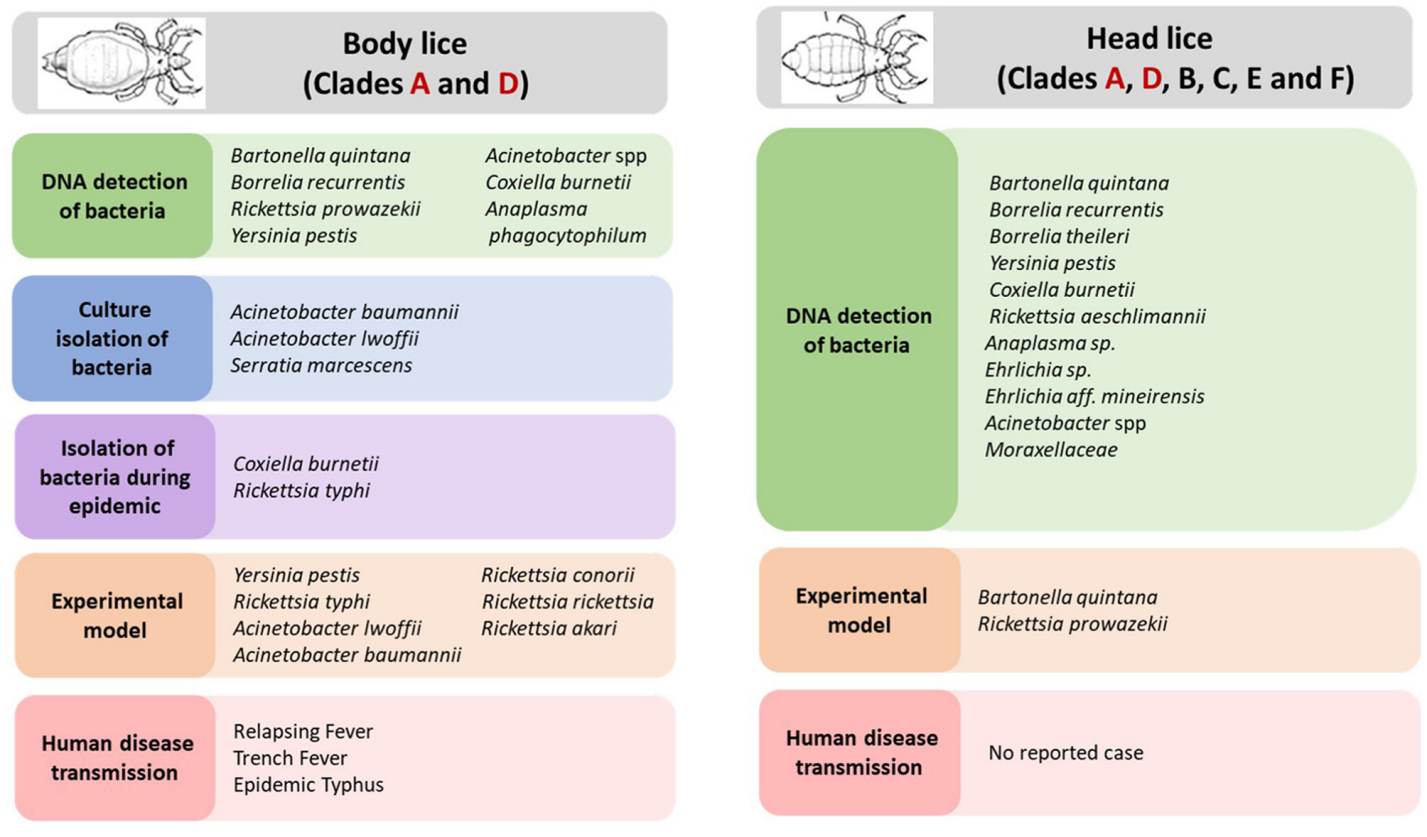
Body and head lice associated bacterial pathogens.

Body louse is the major vector of three humans pathogenic bacteria, which are: *Rickettsia prowazekii, Borrelia recurrentis*, and *Bartonella quintana* (Figure 2) (Raoult and Roux, 1999; Brouqui, 2011). All these three bacterial pathogens have genomes that are smaller in size than their free-living close relatives (Veracx and Raoult, 2012). Body lice acquire infection during blood meals from an infected patient, carrying this infection for the remainder of their short life (Raoult and Roux, 1999). The transmission of these infections to uninfected people occurs through the feces or crushed bodies of infected lice, that contaminate the bite sites, conjunctiva, mucous membranes or microlesions of the skin (Raoult and Roux, 1999).

*Rickettsia prowazekii* is the causative agent of epidemic typhus, which caused significant health problems in times of war and social disorder (Raoult and Roux, 1999; Bechah et al., 2008). Despite the effectiveness of antibiotic treatment, the disease remains a serious health threat because it can reoccur at any time (Bechah et al., 2008; Brouqui, 2011). Indeed, healed patients can keep the infection from it for life and, under stressful conditions, the outbreak can occur as milder typhus, Brill-Zinsser's disease, which could be a source of new outbreaks if lice reappear (Bechah et al., 2008; Brouqui, 2011). Furthermore, since the infection with *R. prowazekii* can occur by inhalation, the bacterium is also classified as a biological selective agent (Bechah et al., 2008).

*Borrelia recurrentis* is a spirochete that causes relapsing fever (Raoult and Roux, 1999). In the past, massive epidemics have affected Africa and Eurasia, but nowadays the disease has persisted, particularly in Ethiopia and in other surrounding countries, and has recently emerged among travelers returning from endemic areas in Europe and North America (Bonilla et al., 2013; Antinori et al., 2016a,b).

*Bartonella quintana*, is well-known as the causative agent of trench fever, but it may also cause clinical manifestations, including chronic bacteremia, bacillary angiomatosis and endocarditis (Raoult and Roux, 1999; Brouqui, 2011). The disease was widespread in France during the Napoleonic Wars (1803–1815) and the two world wars (Raoult and Roux, 1999; Raoult et al., 2006). Currently, it is considered as a re-emerging pathogen in poor countries, and among homeless populations in the developed countries (Brouqui, 2011). It has long been agreed that *B. quintana* was transmitted only by body louse on humans, which is thought to be the sole reservoir. However, some human cases of *B. quintana* infection have been associated with kittens and flea-infested kittens (Raoult et al., 1994; Drancourt et al., 1996). The bacterium was also recovered from a cat's dental pulp (La et al., 2005) and in cat fleas collected in France (Rolain et al., 2003). These data suggest that cats may serve as reservoir hosts for *B. quintana* and may follow a transmission mode similar to that described for *B. henselae* in cat-scratch disease. In addition, *Pedicinus obtusus* lice and their macaque host have also been implicated in the transmission cycle of *B. quintana* (Li et al., 2013; Sato et al., 2015). The role of head louse as additional vector was also raised and discussed thereafter.

The combined evidence from laboratory and epidemiological studies strongly implicates body lice as a vector of *Yersinia pestis*, the causal agent of plague (Houhamdi et al., 2006; Piarroux et al., 2013). Body lice could be involved in plague pandemics that would better match the epidemiology of louse borne infections, this hypothesis has been established by paleomicrobiological studies (Raoult, 2016).

Moreover, the potential role of body louse as vectors for other pathogenic bacteria has also been suspected (Figure 2). Indeed, studies showed that experimentally infected body lice have the potential to acquire, to sustain and to transmit *R. conorii* (Mediterranean spotted fever, Indian tick typhus), *R. rickettsii* (Rocky Mountain spotted fever), which are both transmitted by ticks, and *R. akari* (rickettsial pox) and *R. typhi* (endemic or murine typhus), which are typically transmitted, respectively, by acari mites and insect fleas (Weyer, 1952; Houhamdi et al., 2003; Houhamdi and Raoult, 2006b). Furthermore, during an outbreak of murine typhus that occurred in northern China and India (Kashmir State), *R. typhi* has been isolated from body lice infesting the sick patients (Liu, 1944; Kalra and Rao, 1951). Initial field observations in East Africa reported that body lice recovered from an area where a Q fever outbreak that had took place 3 months before had been able to transmit *Coxiella burnetii* to guinea pigs (Giroud and Jadin, 1954; Babudieri, 1959). Subsequently, it was reported that *C. burnetii* infection of body lice could occur under experimental conditions (Babudieri, 1959). Other bacteria species, such as *Serratia marcescens Acinetobacter baumannii* and *A. lwoffii* have been linked with body lice, assuming that body lice may also transmit these agents (La Scola et al., 2001; Houhamdi and Raoult, 2006a).

Together, these studies suggested that body lice may carry a broad spectrum of pathogens bacteria. In addition, as blood-feeder insect lice are predisposed to absorb many other types of microorganisms, including viruses and hemoparasites, when they feed on their human host (such as *Babesia* or *Plasmodium*). Theoretically, it is possible that lice can transmit any of these agents, if they are ingested with blood meal and if they are able to survive in the insect's midgut (Raoult and Roux, 1999). Moreover, the very reduced genome of human lice lacks many genes associated with immune response, odorant and gustatory receptors and detoxifying enzymes (Kirkness et al., 2010; Lee et al., 2010; Previte et al., 2014). The reduced number of defense mechanisms may facilitate the louse infection by different microorganisms. Furthermore, a recent study suggested that body louse phagocytes might host microbes and could be reservoirs for several unidentified pathogens (Coulaud et al., 2015). Therefore, the role of body lice as vectors of these microorganisms is an interesting research topic that deserves to be addressed in future studies.

### Head Louse Associated Pathogens

In recent decades, there has been a growing recognition that head lice are vectors of pathogens, which has changed the long-established paradigm that only body lice are vectors of disease. Although it is currently agreed that body lice are the most powerful vectors of pathogens, the potential of head lice as disease vector is not yet fully understood.

Recently, several studies have performed comparative analyses in the immune responses following the bacterial challenge using *in vitro*-rearing system and have shown that body lice relative to head lice show a significant reduction in immune responses, particularly at the onset of the immune challenge (Kim et al., 2011, 2012, 2017; Previte et al., 2014). In one study in particular, Kim et al. (2017) demonstrated that, as in the case of body lice, head lice can withstand a persistent charge of *B. quintana* infection for several days following the acquisition in a bloodmeal and spread in their feces viable bacteria. However, the rates of proliferation in the gut and numbers of viable *B. quintana* in the feces were significantly lower in head lice compared to body lice (Kim et al., 2017). For example, in body lice, the *B. quintana* proliferation rate in the gut was significantly higher (2.0–12.1-fold) over time when compared with head lice until 12 days post-challenge. The viability index of *B. quintana* in the feces over the entire 11 days post-challenge was 6.4–10.6-fold higher in body lice compared to head lice (Kim et al., 2017). The reduction of the immune response in body lice may be partly the consequence for their increased competence as a vector, compared to head lice (Kim et al., 2011, 2012, 2017; Previte et al., 2014), which develop a more effective immune response to restrict bacterial replication.

Indeed, following *B. quintana* oral challenge, the transcriptional analysis of the immune response of the alimentary tract of body lice, revealed a reduction in the level of transcription of the main immune genes, including peptidoglycan recognition protein, defensins, and defensin 1, when compared to head lice. How this transcription repression occurs in body lice remains currently unknown (Kim et al., 2012, 2017). Kim et al. (2017) suggested that this regulation may be a consequence of regulatory changes in the epigenetic pathways and in the post-transcriptional miRNA-mediated factors (Kim et al., 2017). The level of reactive oxygen species, which is thought to be one of the key mediators of antimicrobial innate immunity defense in the insect gut (Molina-Cruz et al., 2008), was also reduced in body lice compared to head lice (Kim et al., 2017). Moreover, the role of endosymbiotic bacteria in the modulation of the host immune responses to pathogenic infections should be investigated (Kim et al., 2017). The impact of endosymbionts to insect antibacterial immune responses was already described in several insects (Eleftherianos et al., 2013). For example, the flies carrying the *Spiroplasma* endosymbiont have been shown to be more susceptible to septic injury with some Gram-negative bacteria, compared to flies lacking the endosymbiont. These results suggest that *Spiroplasma* endosymbionts are able to confer antibacterial immunity against certain bacterial pathogens in flies (Herren and Lemaitre, 2011).

Moreover, the fact that body and head lice have distinct ecological niches and have distinctly different feeding patterns, are additional factors that might influence the transmission of pathogens by lice. Body lice live in clothing where they must face the movements of the host's body to access blood, so they are under increased stress compared to head lice (Raoult and Roux, 1999; Veracx and Raoult, 2012). The effects of stress on immunity are well-known in insect vectors (Muturi et al., 2011) and may cause alterations that may lead to the reduced innate immune response observed in body lice against pathogenic bacteria. Similarly, the body lice are known to ingest large blood meal as compared to head lice (Brouqui, 2011; Veracx and Raoult, 2012). Absorption of larger meals of infectious blood may result in more bacteria entering the louse midgut, and therefore, a possible increase in infection.

Under laboratory conditions, head lice can also transmit *R. prowazekii*. The first evidence was reported by Goldberger and Anderson, who have successfully transmitted typhus to naïve rhesus macaques by subcutaneous inoculation of infected crushed head lice, recovered from patients with epidemic typhus (Goldberger and Anderson, 1912). Subsequently, this finding was confirmed by Murray and Torrey (1975) who showed that head lice feeding on rabbits infected with *R. prowazekii* can be easily infected and spread virulent organisms in their feces, thus demonstrating that head lice have the potential to transmit pathogenic bacteria when placed under favorable epidemiological conditions (Murray and Torrey, 1975). In addition, it has been proposed that this louse could also be implicated in the transmission and maintenance of this pathogen in the nature (Robinson et al., 2003).

In recent years, several pathogenic bacteria DNA have been increasingly reported in head lice in many parts of the world (Figure 2). Detailed results on the epidemiological studies are reported in Table 1. For instance, *B. quintana* was the most frequently found in head lice belonging to clades A, D, E, and C. Furthermore, it is important to underline that, except one reported case of its detection in lice collected from school children (Eremeeva et al., 2017), all other cases occurred on lice infesting deprived populations living in poverty and lacking standard medications (Table 1). An interesting study of head lice collected from the rural population of Senegal, where several cases of trench fever have occurred, showed that several head lice were positive for *B. quintana*. These findings, together with the absence of body lice from this area for more than 30 years, strongly implicate head lice as the principal acting in maintaining the cycle of transmission of *B. quintana* among the population concerned (Diatta et al., 2014).

**Table 1:** Bacteria species found in head lice using molecular methods.

**Pathogen**	**Lice tested (% positive)**	**Louse-clade**	**Collection year**	**Population**	**Country**	**Co-occurrence of body louse**	**Comment**	**References**
*B. quintana*	21 (9.5%)	–	2002	Homeless and school children	Nepal	Yes	All lice collected from school children were *B. quintana* negatives	Sasaki et al., 2006
	15 pools (20%)	–	2007–2008	Homeless persons	California-USA	Not all	–	Bonilla et al., 2009
	16 pools (37.5%)	–	2008–2010, 2012	Homeless persons	California-USA	Not all	–	Bonilla et al., 2014
	3 pools nits (100%)	–	–	Homeless persons	France	No	Attempt to cultivate *B. quintana* from these nits was failed	Angelakis et al., 2011b
	35 (17.1%)	A and D	2010	Persons living in a highly plague-endemic area	Congo RDC	Yes	–	Piarroux et al., 2013; Drali et al., 2015b
	35 (2.85%)	–	2011	Patients with louse-borne relapsing fever	Ethiopia	Yes	–	Boutellis et al., 2013b
	65 pools (9.2%)	–	2010	Street beggars (in poorer regions)	Ethiopia	–	More pools of head lice were found positive than body lice	Cutler et al., 2012
	271 (7%)	C	–	Persons living at locations at different altitudes	Ethiopia	Not all	*B. quintana* in head lice was positively linked to altitude (>2,121 m). At this altitude all body lice were *B. quintana* negatives	Angelakis et al., 2011a
	274 (6.93%)	A and E	2010–2011	Persons living in poor conditions	Senegal	No	–	Boutellis et al., 2012
	381 (0.52)	A	2011	Rural community	Senegal	–	–	Sangaré et al., 2014
	148 (1.3%)	A	2011	Rural villagers living in poor conditions	Senegal	No	Co-occurrence of trench fever cases with absence of body lice for more than 30 years in the studied area	Diatta et al., 2014
	75 (2.66)	A	2010–2011	Rural community with low income	Madagascar	–	–	Sangaré et al., 2014
	600 (0.5%)	E	2013	Rural villagers living in poor conditions	Mali	No	Apparently healthy individuals, low socioeconomic level	Amanzougaghene et al., 2017
	168 (10.3%)		2013–2015	School children	Georgia-USA	No	The *kdr*-permethrin resistance (T917I mutation) was detected in 99.9% of 168 lice tested	Eremeeva et al., 2017
*B. recurrentis*	35 (22.8%)	–	2011	Patients with louse-borne relapsing fever	Ethiopia	Not all	4 of 5 patients were co-infested with body louse	Boutellis et al., 2013b
	630 (1.6%)	A	2015	Pygmy populations, living in poor conditions	Republic of Congo	No	–	Amanzougaghene et al., 2016a
*Y. pestis*	35 (2.86%)	A	2010	Persons living in a highly plague-endemic area	Congo RDC	Yes	–	Piarroux et al., 2013; Drali et al., 2015b
*B. theileri*	630 (0.16%)	A	2015	Pygmy populations, living in poor conditions	Republic of Congo	No	–	Amanzougaghene et al., 2016a
*C. burnetii*	600 (1.16%)	E	2013	Rural villagers living in poor conditions	Mali	No	MST genotype 35 and new genotype (genotype 59)	Amanzougaghene et al., 2017
	37 (8.10%)	E	2016	Niger's refugees arriving in Algeria, living in poor conditions	Algeria	No	–	Louni et al., 2018
*R. aeschlimannii*	600 (0.6%)	E	2013	Rural villagers living in poor conditions	Mali	No	Apparently healthy individuals, low socioeconomic level	Amanzougaghene et al., 2017
*Anaplasma*	600 (0.3%)	E	2013	Rural villagers living in poor conditions	Mali	No	Potential new species	Amanzougaghene et al., 2017
*Ehrlichia*	600 (2.3%)	E	2013	Rural villagers living in poor conditions	Mali	No	*E. aff. mineirensis* and potential new species	Amanzougaghene et al., 2017
*Acinetobacter*	288 (33%)	–	2008–2009	School children	France	–	–	Bouvresse et al., 2011
	115 (47%)	A and C	–	Healthy individuals	Ethiopia	Yes	13 of 23 lice sequenced were *A. baumannii*	Kempf et al., 2012
	275 (3.62%)	A and C	2013–2014	School children	Thailand	No	Species: *A. baumannii, A. radioresistens*, and *A. schindleri*	Sunantaraporn et al., 2015
	630 (31.1%)	A, C, and D	2015	Pygmy populations, living in poor conditions	Republic of Congo	No	Species: *A. junii, A. ursingii, A. baumannii, A. johnsonii, A. schindleri, A. lwoffii, A. nosocomialis*, and *A. towneri*	Amanzougaghene et al., 2016a
	52 (80.8%)	–	2013–2015	School children	Georgia-USA	No	–	Eremeeva et al., 2017
	235 (11.5%)	A, B, and E	2015–2016	Middle-class suburban families	France	No	*A. baumannii, A. calcoaceticus, A. nosocomialis, A. junii*, and 2 potential new species (*Candidatus* Acinetobacter Bobigny-1 and 2)	Candy et al., 2018a
	64 (27%)	A and B	2014	School children	Algeria	No	*A. baumannii, A. johnsonii*, and *A. variabilis*	Mana et al., 2017
	37 (46.94%)	E	2016	Niger's refugees arriving in Algeria	Algeria	No	*A. baumannii*	Louni et al., 2018
*Moraxellaceae*	630 (0.95%)	A	2015	Pygmy populations, living in poor conditions	Republic of Congo	No	New species	Amanzougaghene et al., 2016a

The DNA of this bacterium was also recovered in head louse nits from homeless individuals in Marseille. This funding strongly support a possible vertical transmission of this pathogen in lice (Angelakis et al., 2011b). A study performed on lice from persons at various locations in Ethiopia revealed that head lice infection by *B. quintana* was linked to high altitude (Angelakis et al., 2011a). Interestingly, the head lice collected from persons living at higher altitudes (>2,121 m) were *B. quintana* positive (24% of 79), while at these altitudes, none of the 63 tested body lice were found to be infected with *B. quintana* (Angelakis et al., 2011a). This finding raises important question about the possible relationship of the head lice vectorial capacity and environmental-ecological factors that may drive force underpinning the transmission potential in some geographic areas.

*Borrelia recurrentis* was detected in clade C head lice from patients suffering with louse-borne relapsing fever living in poor regions from Ethiopia, and more recently in head lice clade A from hunter-gatherer pygmy individuals in the Republic of the Congo (Boutellis et al., 2013b; Amanzougaghene et al., 2016a). The *Y. pestis* DNA was also found in clade A head lice infesting individuals from a highly endemic plague area in the eastern Congo (Piarroux et al., 2013; Drali et al., 2015b). Other human pathogenic bacteria, which are not usually associated with lice transmission, such as *C. burnetii, R. aeschlimannii*, and potential new species from the *Anaplasma* and *Ehrlichia* genera were also detected in head lice (Amanzougaghene et al., 2017). Moreover, the DNA of Ixodid hard tick-associated *B. theileri*, which is not known to infect humans, was also detected in one head louse infesting an African pygmy from the Republic of Congo (Amanzougaghene et al., 2016a). As head lice feed only on human blood, the authors suggested that the acquired louse infection would be from the blood of patients with ongoing bacteremia (Amanzougaghene et al., 2016a). However, whether this spirochete is able to infect human is currently not known and requires further investigation.

Head louse infection with all of the above pathogenic bacteria most likely occurs among the most vulnerable and poor populations living in poverty and poor hygiene, the same conditions that usually lead to the proliferation of body louse ecotype and the emergence of louse-borne diseases (Raoult and Roux, 1999; Brouqui, 2011). This point of view is also supported by the fact that most studies on head lice infesting schoolchildren and populations living in more hygienic conditions have failed to detect these pathogenic bacteria (Fournier et al., 2002; Bouvresse et al., 2011; Sunantaraporn et al., 2015; Candy et al., 2018a).

However, head lice infection with *Acinetobacter* is an exception, as several species have been detected with high prevalence in head lice, as well as in body lice everywhere they have been sought, reflecting the ubiquitous occurrence of these bacterial species (Bouvresse et al., 2011; Sunantaraporn et al., 2015; Amanzougaghene et al., 2016a; Candy et al., 2018a). This widespread infection of human lice suggests that they could be a preferential host for these bacteria. Nevertheless, it remains to be determined whether these strains of *Acinetobacter* found in lice are the same as those causing infections in humans (Amanzougaghene et al., 2016a; Candy et al., 2018a). In addition, the susceptibility of these isolates to antibiotics is currently unknown. In one study, Vallenet et al. (2008) showed that phenotypic and genotypic characteristics of the human multidrug resistant isolate *A*. *baumannii* AYE significantly differ from the SDF strain isolated from body lice (Vallenet et al., 2008). Further studies are warranted to compare both phenotypic and genotypic characteristics, as well as antimicrobial susceptibility of louse derived *Acinetobacter* isolates. Greater attention is given to extra-hospital reservoirs of these opportunistic bacteria and their potential involvement in emerging human community-acquired infections, as drug-resistant strains worldwide are increasingly being identified (Eveillard et al., 2013).

Based on the combined evidence of both epidemiological and laboratory studies, we believe that head lice can transmit disease to their human host under favorable epidemiological conditions, although its vectorial capacity is weaker compared to body lice. Therefore, given the scale of head louse infestations around the world and the emergence and spread of insecticides resistance, this pest is warranting more attention as a serious public health problem. For instance, epidemiological studies on louse specimens collected worldwide to investigate the diversity of pathogenic bacteria associated with head lice, but also for body lice, are missing. Regarding experimental models, the vectorial competence of lice should be investigated for pathogenic bacteria, such as *C. burnetii*, and other pathogenic bacteria, which are not usually associated with lice.

## Control of Lice Infestations and Evolution of Insecticide Resistance

### Therapeutic Options for Pediculosis Treatment

There are many therapeutic options for pediculosis, including chemical insecticides, topically applied physical agents (Table 2), herbal formulations and mechanical methods (combs and heating devices) (Bonilla et al., 2013; Feldmeier, 2014; Sangaré et al., 2016a). However, the recourse to chemical insecticides with an insect neurotoxic mode of action is still the method of choice and the most extensively used approach (Durand et al., 2012; Clark et al., 2013). These include organochloride (lindane), organophosphates (malathion), carbamates (carbaryl), pyrethrins (extract of chrysanthemum), and pyrethroids (synthetic derivates of pyrethrins: permethrin, phenothrin, and deltamethrin) (Table 2) (Durand et al., 2012; Bonilla et al., 2013; Clark et al., 2013). Among them, malathion and permethrin remain the most widely used pediculicides since their introduction on the market in 1971 and 1992, respectively (Durand et al., 2012; Clark et al., 2013).

**Table 2:** Main Therapeutic options for pediculosis treatment.

**Pediculicide**	**Class**	**Mechanism of action**	**Adulticide/ovicidal activities**	**Documented adverse health effect**	**Documented resistance in lice**	**References**
**PEDICULICIDE WITH A NEUROTOXIC MODE OF ACTION**						
DDT, dichlorodiphenyltrichloroethane	Organochloride	Opening of sodium ion channels in neurons	Yes/yes	Toxic	Yes	Durand et al., 2012; Bonilla et al., 2013
Lindane	Organochloride	Inhibition of c-aminobutyric acid- gated chloride channel	Yes/yes	Toxic	Yes	Durand et al., 2012; Bonilla et al., 2013
Natural pyrethrins	Chrysanthemum extract	Delayed repolarization of voltage-gated	Yes/no	Minor	Yes	Bonilla et al., 2013
Permethrin, synthetic pyrethrin	(+)-3-phenoxybenzyl 3-(2,2-dichlorovinyl)-2,2, dimethyl cyclopropan carboxylate	The same as natural pyrethrins	Yes/no	Minor	Yes	Durand et al., 2012; Bonilla et al., 2013; Clark et al., 2013
Malathion	Organophosphate	Irreversible inhibition of acetylcholinesterase	Yes/no	Minor	Yes	Durand et al., 2012; Bonilla et al., 2013; Kwon et al., 2014
Carbaryl	Carbamate	Irreversible inhibition of acetylcholinesterase	Yes/no	Moderate to very toxic	Yes	Durand et al., 2012; Bonilla et al., 2013
Ivermectin^*^	Macrocyclic lactone	Binding to glutamate-gated chloride ion channels	Yes/no	None to minimal	Yes	Chosidow et al., 2010; Clark et al., 2013; Diatta et al., 2016
Spinosad	Macrocyclic lactone	Overstimulates nerve cells by acting like acetylcholine	Yes/yes	Minor	No	Aditya and Rattan, 2012; Feldmeier, 2014
**PEDICULICIDE WITH PHYSICAL MODE OF ACTION**						
Dimeticone	Synthetic silicone oils	Work by occlusion	Yes/yes	Low	No	Durand et al., 2012; Burgess et al., 2013; Feldmeier, 2014
Isopropyl myristate	Ester	Work by occlusion or by dissolving cuticle wax	Only head lice tested	Minimal	No	Feldmeier, 2014
1,2-octanediol	Detergent	Dehydration by reducing the ability of louse to prevent water loss through the cuticle	Yes/no	Minimal	No	Burgess et al., 2012; Feldmeier, 2014; Burgess and Silverston, 2015
Benzyl alcohol	Aromatic alcohol	Asphyxiates lice by “stunning” the spiracles open	Yes/no	Minimal	No	Meinking et al., 2010

* *Ivermectin is the only pediculicide used on topical and oral administrations whereas the other pediculicides are only available through topical applications*.

Unfortunately, the wider use of conventional pediculicides has resulted in the emergence and rapid propagation of resistant lice populations in many regions of the world (Durand et al., 2012; Clark et al., 2013). This has prompted research into the development of compounds with other modes of action. Ivermectin and spinosad seem to be the most hopeful new pediculicides. They have generated interest in their new neurotoxic modes of action; have low mammalian toxicity and relatively low cross-resistance with commonly used conventional pediculicides (Chosidow et al., 2010; Aditya and Rattan, 2012; Bonilla et al., 2013; Clark et al., 2015). In addition, ivermectin is the only drug currently used for oral treatment, and its highly effectiveness was clinically approved for both louse ecotypes treatment (Foucault et al., 2006; Chosidow et al., 2010), although empirically noted ivermectin resistance has started to be reported in the field in Senegal (Diatta et al., 2016).

There is also growing interest in the use of natural products such as pediculicides based on plant-derived essential oils (eucalyptus oil and tea tree oil) or with a purely physical mode of action, such as dimeticone and benzyl alcohol (Meinking et al., 2010; Burgess et al., 2013; Candy et al., 2018b), but little attention has been given to their clinical evaluation for effectiveness, even though some of them are already commercialized.

### Insecticide Resistance

Insecticide resistance leading to treatment failure is considered a crucial factor in the increasing incidence of head lice infestations (Durand et al., 2012; Bonilla et al., 2013). Resistance is an established trait that an insect pest acquires over time through selective pressure from continuous or inappropriate insecticide use (Durand et al., 2012). The recently sequenced body louse genome provides a unique opportunity to address fundamental issues related to the molecular mechanisms that determine the insecticides resistances, which is fundamental to ensure the longest possible active lifespan of existing insecticides and to accelerate the achievement of sustainable, new, and effective strategies to control pest infestations (Kirkness et al., 2010; Clark et al., 2015).

Possible resistance mechanisms include knockdown-resistance in the case of permethrin, which is the result of three-point mutations (M815I, T917I, and L920) within the α-subunit gene of the voltage-gated sodium channel, and enhanced activity of a carboxylesterase enzyme was found to be chiefly responsible for the resistance to malathion (Clark, 2009; Kwon et al., 2014). Recently, a researcher from our team documented the first field resistance to ivermectin observed in head lice recovered in rural populations from Senegal (Diatta et al., 2016). Genetic analysis of these lice by targeting GluCl gene, which is the primary target-site of ivermectin and already known to be implicated in resistance of arthropods and nematodes, revealed the presence of three relevant non-synonymous mutations (A251V, H272R, and S46P) that may be responsible for the treatment failure (Amanzougaghene et al., 2018a).

In addition, in another study, a proteomic comparison of laboratory-sensitive lice (wild type) and ivermectin-selected resistant lice found that a complexin was most significatively suppressed in resistant lice. A complexin is a neuronal protein that is known to be one of the main regulators of neurotransmitter release. DNA-mutation analysis revealed that some complexin transcripts from resistant lice gained a premature stop codon. The association between complexin and ivermectin-resistance was further confirmed by RNA-interfering and found that the knocking down complexin expression induces resistance to ivermectin in the susceptible lice. All together, these results provide a convincing evidence that complexin plays a significant role in regulating ivermectin resistance in the body louse and represents the first evidence that links complexin to insecticide resistance (Amanzougaghene et al., 2018b). Although, how this resistance effect is mediated requires further elucidation.

### New Therapeutic Approaches

#### Symbiotic Therapy

This approach incited great interest for potential applications in public health entomology (Sassera et al., 2013) and has the advantage to target the bacteria susceptible to antibiotics (Sassera et al., 2013). Since the *Pediculus* lice' primary endosymbiont, *Candidatus* Riesia pediculicola, is essential to the metabolism of their louse host, related to its ability of synthesizing B-group vitamins, the harmful effect exerted by the antibiotic treatment should have repercussions on the host (Kirkness et al., 2010; Sangaré et al., 2016a). A first case report showed that antibiotic treatment (trimethoprim and sulfamethoxazole), administered to treat a respiratory infection in a 12-year-old girl, had the side effect of head lice death (Shashindran et al., 1978). Subsequently, studies conducted by Sangaré et al. (2015) demonstrated the effectiveness of this therapy under laboratory conditions and showed that antibiotics (such as doxycycline, erythromycin, rifampicin, and azithromycin) kill lice via their direct activity on their symbiotic bacteria. In addition, the combination of this antibiotic with ivermectin has proven to be very effective compared to ivermectin alone in the treatment and prevention of body lice (Sangaré et al., 2016b). This approach could be employed to eradicate lice and could potentially retard the apparition of resistance to ivermectin (Sangaré et al., 2016b). This approach is a promising therapy but has not yet been the subject of field studies.

#### RNAi-Based Insecticides

RNA interference (RNAi) is a promising and safe environmentally friendly method for insect control (Das et al., 2015). This technology is initiated by the presence of a small interfering RNA (siRNA) or double-stranded RNA (dsRNA) to trigger post-transcriptional gene silencing (Das et al., 2015). RNAi can induce mortality, create phenotypes that are beneficial for insect control and prevent insecticide resistance in insect pests (Gordon and Waterhouse, 2007; Airs and Bartholomay, 2017). Currently, the greater potential of this technology for successful future management of pest insects is widely recognized and holds great promise (Baum et al., 2007; Airs and Bartholomay, 2017). Therefore, it is exciting to consider its role in lice control as a promising alternative to chemical insecticides, to specifically to suppress the expression of essential genes that lead to death (Scott et al., 2013). Two lines of evidence support its potential use as control strategy in lice. First, the analysis of body louse genome has been shown to contain the necessary genes for RNAi machinery (Pittendrigh et al., 2013). Subsequently, the studies conducted on both body and head lice confirmed that the injection of dsRNA to these two ecotypes can efficiently repress the expression of target genes (Yoon et al., 2011; Kwon et al., 2014). A second beneficial of using this technology in *Pediculus* lice is the lack of gene redundancy in its small genome. Thus, a smaller set of genes can be investigated to identify which one is essential for a given biological process (Pittendrigh et al., 2013). A priority for the future should focus on the identification of effective target genes for RNAi and, subsequently, on the exploration of delivery methods using field-proven applications (Pittendrigh et al., 2013). Finally, although technically realizable, it remains to be defined whether such strategies could comply with the requirements of regulatory authorities and whether an economically attractive strategy can be implemented to use RNAi approach to fight against lice (Pittendrigh et al., 2013).

## Concluding Remarks and Future Perspectives

In the twenty-first century, human lice infestation remains widespread all over the world. Surprising new discoveries into the biology, epidemiology, and the evolutionary history of lice, their bacterial disease agents and control strategies have further stimulated a renewal of interest in this bloodsucking insect. In recent years, knowledge about lice has evolved, with the sequencing of the body lice genome and the development of transcriptomes of body and head lice. However, functional and comparative genomics of the fundamental aspects of lice biology is still in its infancy and many aspects are not yet well-understood and remain to be discovered.

Additional efforts will be necessary to shed the light on lice biology. Whole genome sequencing of head lice belonging to different clades with integration of high-throughput technologies to study global changes in mRNA transcription, translation and computational approaches, will accelerate the addressing of important biological questions, identification, and exploitation of new target genes of this insect vector, insecticide discovery, as well as to develop novel therapies. Although our knowledge of the vector competence of head lice is increasing, there is still a need to explore factors that can influence the difference observed between body and head lice, such as the influence of the immune response and microbiota (especially the role of endosymbiotic bacteria). Such factors, once addressed, will provide us with a better understanding of effective lice control and prevention strategies for re-emerging diseases. Finally, because *P. humanus* is one of the oldest parasites of human which carries a written record of our past in its DNA, integrating phylogenomic and genomic population patterns in lice will provide a more complete picture of the evolution of this parasite and clarify additional events in our evolutionary history.

## Author Contributions

NA, OM, FF, and DR conceived the paper. NA wrote the paper.

### Conflict of Interest

The authors declare that the research was conducted in the absence of any commercial or financial relationships that could be construed as a potential conflict of interest.
